# Effect of targeted vs standard fortification of breast milk on growth and development of preterm infants (≤ 32 weeks): study protocol for a randomized controlled trial

**DOI:** 10.1186/s13063-020-04841-x

**Published:** 2020-11-23

**Authors:** Joanna Seliga-Siwecka, Anna Chmielewska, Katarzyna Jasińska

**Affiliations:** 1 Neonatal and Intensive Care Department, The Medical University of Warsaw, 2 Karowa Street, 00-315 Warsaw, Poland; 2 Department of Clinical Sciences, Umeå University, Umeå, Sweden

**Keywords:** Preterm, Very low birth weight, Enteral nutrition, Breast milk, Fortification, Growth

## Abstract

**Background:**

Human milk is recommended for all very low birth weight infants. Breastmilk is highly variable in nutrient content, failing to meet the nutritional demands of this group. Fortification of human milk is recommended to prevent extrauterine growth retardation and associated poor neurodevelopmental outcome. However, standard fortification with fixed dose multicomponent fortifier does not account for the variability in milk composition. Targeted fortification is a promising alternative and needs further investigation.

**Methods:**

This randomized controlled trial will recruit preterm infants (≤ 32 weeks of gestation) within the first 7 days of life. After reaching 80 ml/kg/day of enteral feeding, patients will be randomized to receive standard fortification (HMF, Nutricia) or targeted fortification (modular components: Bebilon Bialka, Nutricia—protein; Polycal, Nutricia—carbohydrates; Calogen, Nutricia—lipids). The intervention will continue until 37 weeks of post-conception age or hospital discharge. Parents and outcome assessors will be blinded to the intervention. The primary outcome measure is velocity of weight, length, and head growth until 36 weeks post-conceptional age or discharge. Secondary outcomes include neurodevelopment at 12 months assessed with Bayley Scale of Development III, repeated at 36 months; body composition at discharge and at 4 months; and incidence of necrotizing enterocolitis, sepsis, retinopathy of prematurity, and bronchopulmonary dysplasia.

**Discussion:**

Targeted fortification has previously been shown as doable in the neonatal intensive care unit context. If it shows to improve growth and neonatal outcome, choosing the targeted fortification as a first line nutritional approach in very low birth weight infants may become a recommendation.

**Trial registration:**

ClinicalTrials.govNCT03775785, Registered on July 2019.

## Administrative information

The order of the items has been modified to group similar items (see http://www.equator-network.org/reporting-guidelines/spirit-2013-statement-defining-standard-protocol-items-for-clinical-trials/).

**Table:** 

Title {1}	Effect of targeted vs standard fortification of breast milk on growth and development of preterm infants (≤ 32 weeks): a randomized controlled trial.
Trial registration {2a and 2b}.	Clinicaltrials.gov (NCT03775785). WHO trial registry items can be found within the body of the protocol.
Protocol version {3}	version 2. 28.02.2019
Funding {4}	This work was supported by Nutricia Research Foundation, grant number RG 4/2018.
Author details {5a}	Joanna Seliga-Siwecka MD, PhD Neonatal and Intensive Care Department, The Medical University of Warsaw, Warsaw, Poland Anna Chmielewska, MD, PhD Department of Clinical Sciences Umeå University, Sweden Katarzyna Jasińska, MD Neonatal and Intensive Care Department, The Medical University of Warsaw, Warsaw, Poland
Name and contact information for the trial sponsor {5b}	The Nutricia Foundation 6 Bobrowiecka street 00-728 Warsaw, Poland
Role of sponsor {5c}	The funder will not have any role during the study execution, analyses, interpretation of the data, or decision to submit results.

## Introduction

### Background and rationale {6a}

#### Premature birth

About 15 million of infants are born premature (< 37 completed weeks of gestation) every year. Prematurity is a leading cause of death in newborns and (after pneumonia) in children up to 5 years of age. Children born premature suffer from long-term health consequences such as unfavourable cardiovascular and metabolic profile, and poorer neurological outcome [1].

In spite of a tremendous improvement in neonatal care during the last decade, nutrition in very premature infants still poses a challenge. The recommended nutritional intakes are often not achieved, and extrauterine growth restriction (EUGR) is common even in developed countries [2]. At achieving term-equivalent age, extremely preterm infants are lighter and shorter, and they have smaller head circumference and less favourable body composition compared to their term peers [3, 4]. Suboptimal nutritional intakes and postnatal growth restriction have long-term negative impact on growth and development and are associated with lower IQ scores at school age (Lucas [5]; Ehrenkranz et al. [6]).

Human milk is considered the best nutrition choice for all very low birth weight (VLBW) infants due to multiple benefits such as improved feeding tolerance, lower rates of necrotizing enterocolitis (NEC) and sepsis, reduced length of hospital stay, and, in a long perspective, improved neurodevelopment and lower risk for hypertension [7].

#### Fortification of human milk

Native human milk does not cover the high nutritional needs of VLBW, and so fortification of both own mother’s milk (OMM) and donor human milk (DHM) is recommended to prevent EUGR and the associated poor neurodevelopmental outcome [6, 8, 9]. Currently, there are three types of human milk fortification.

*Standard fortification* (SF) with a fixed dosage compound product is based on assumed nutrient content of OMM or DHM without accounting for nutrient concentrations in preterm human milk. Human milk however is highly variable in the nutrient content, both between the mothers and between the samples from the same mother [10, 11]. A recent study suggested that not taking this variability into account leads to inadequate intake in about 25–40% VLBW due to low content of protein and energy [12].

*Adjustable fortification* is based on supplementing naïve human milk with protein. This approach uses blood urea nitrogen (BUN) levels as a surrogate of metabolic response to the fortification. In a retrospective study, preterm infants receiving adjusted fortification had higher intakes of protein, better growth pace, and higher developmental scores at 18 months compared to infants on standard fortification [13].

*Tailored/targeted fortification* (TF) accounts for variability of native human milk content. Milk analysis is performed on regular basis to determine its macronutrient content. Based on the results, protein, fat, and carbohydrate are added to milk as modular products with the aim to achieve the recommended concentrations [14]. Feasibility and safety of TF was shown in a pilot study by Rochow et al. [12]. The same group conducted a trial including 157 VLBW, which showed that targeted fortification of human milk improved growth velocity (conference abstract/personal communication) [15]. Long-term effects of targeted fortification, such as neurodevelopment and body composition, remain to be studied.

The current randomized trial will compare targeted fortification with standard fortification (the method most commonly used in clinical practice) and its effect on growth, body composition, and development in premature infants born < 32 weeks of gestation.

### Objectives {7}

#### Research hypothesis

Tailored fortification of enteral nutrition improves weight gain velocity of preterm infants born at ≤ 32 weeks of gestation compared to standard fortification.

#### Study objectives

##### Primary objective

The primary objective is to determine if tailored compared to standard fortification of enteral nutrition improves weight gain velocity of preterm infants born at ≤ 32 weeks of gestation.

##### Secondary objectives

*Key secondary objectives*

The key secondary objectives are to determine the following anthropometric parameters in preterm infants born at ≤ 32 weeks of gestation at discharge and at 4 months:

- Feeding tolerance
- Velocity of weight gain
- Length and head growth
- Body composition (by air displacement plethysmography, PeaPod)

*Other secondary objectives*

1. Neurodevelopmental outcome:
  12 months corrected age: BSID-III (Bayley Scale of Development Third Edition)
  3.5 years of corrected age: BSID and WPSSI-IV (Wechsler Preschool and Primary Scale of Intelligence, Fourth Edition IV)
2. Behavioural problems: parental assessment by CBCL (Child Behaviour Check List) at 3.5 years corrected age
3. To compare the incidence of NEC, retinopathy of prematurity (ROP), and bronchopulmonary dysplasia (BPD) between both study groups

### Trial design {8}

The trial is designed as a randomized observer and patient blinded controlled multicentre superiority trial with two parallel groups with 1:1 allocation ratio.

## Methods: participants, interventions, and outcomes

### Study setting {9}

The study will be carried out in three different sites (2 separate neonatal departments within the Medical University of Warsaw, and the Neonatal Department of the Wroclaw Medical University). All study sites are level III teaching hospital with approximately 2500–3000 (100 ≤ 32 weeks of gestation) deliveries per year. The local protocol is based on standard fortification of OMM and DHM. However, the staff is willing to implement new procedures in order to improve neonatal outcome. This should improve trial recruitment and adherence. The current OMM feeding rate during hospital stay in infants born at < 32 gestational age (GA) is 80%.

### Eligibility criteria {10}

All parents of infants born at less than 32 weeks of GA and admitted to the participating units will be approached by one of the research team members within the first week of life (as full enteral feeding is usually reached at a minimum of 7 days of life). Recruitment will take place between June 2019 and December 2021. After obtaining written consent for participating in the trial, the patient’s medical record number (MRN) will be immediately registered on a secure web-based platform and demographic data will be recorded.

#### Inclusion criteria

Patients eligible for the trial must comply with all of the following at randomization:

1. Gestational age at birth ≤32 weeks
2. Enteral feeding of at least 80 ml/kg/day
3. 50% donor or maternal milk-based enteral feeding
4. Parenteral/legal guardian consent

#### Exclusion criteria

1. Formula feeding
2. Small for gestational age (birth weight < 3rd percentile)
3. Presence of congenital abnormalities, which increase the risk of NEC
4. NEC
5. Withdrawal of feeding > 7 days
6. Sepsis
7. Death

### Who will take informed consent? {26a}

All parents of infants born at less than 32 weeks of gestation and admitted to one of the study sites will be approached by one of the research team members within the first 7 days of life. He/she will provide oral and written information about the study. Parents of the patient will then be able to have an informed discussion with the attending physician and/or study personnel. Research team members will obtain written consent from the parents willing to participate in the trial. Information consent forms and information sheets will only be provided in Polish for all parents. Given the limited diversity of our population, we will not recruit newborns of foreign parents unless they speak Polish on a level allowing full understanding of the study conduct.

### Additional consent provisions for collection and use of participant data and biological specimens {26b}

We do not plan to collect samples for ancillary studies.

### Interventions

#### Explanation for the choice of comparators {6b}

SF is based on the assumption that all human milk has a protein level of 1.5 g/dl. Human milk however is highly variable in the nutrient content, both between the mothers and between the samples from the same mother [10, 11]. A recent study suggested that not taking this variability into account leads to inadequate intake in about 25–40% VLBW due to low content of protein and energy [12]. Nonetheless, it is the most widely used strategy for human milk fortification, and thus, its choice as a comparator is logical.

#### Intervention description {11a}

**Fig. 1:**
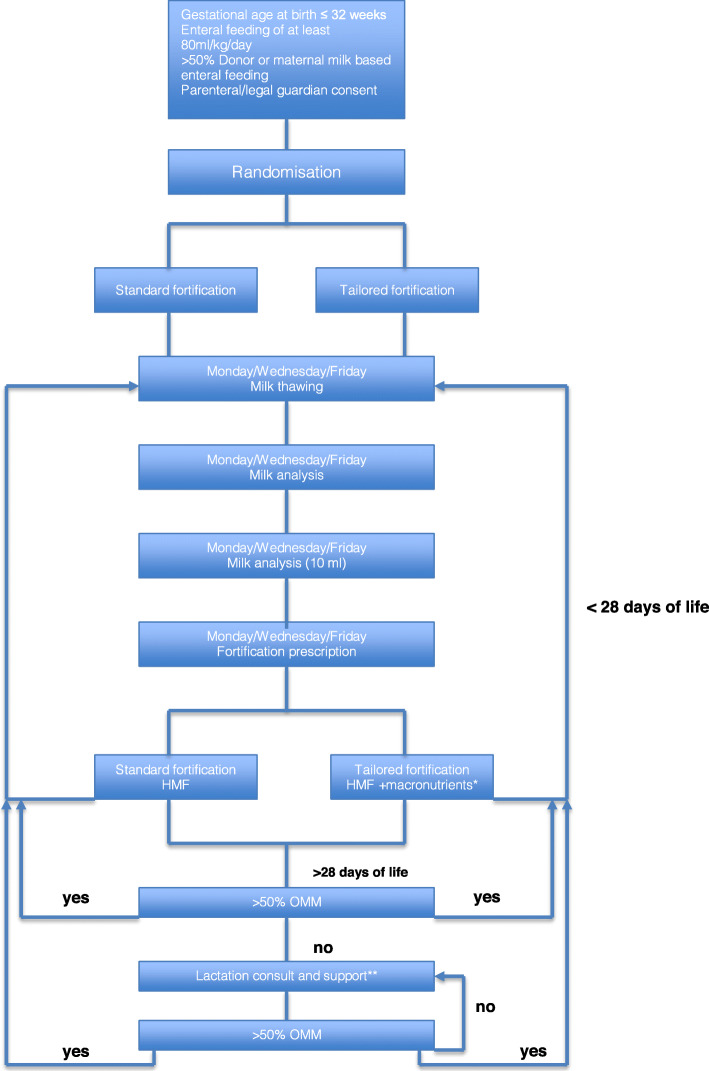
Algorithm for tailored enteral nutrition. *Additional amount of fat, protein, and/or carbohydrate required to achieve target levels of macronutrients will be calculated as addition = ESPGHAN recommendations (OMM/HDM + increment by routine fortification). **One-to-one CLC support, followed by weekly lactation re-evaluation. If OMM amount is less than 20 ml/day for ethical reasons, it will not be submitted for analysis and fortification will be continued as per most recent report. *To enhance feeding tolerance at the start of the intervention, TF will be gradually introduced in a stepwise manner over a 3-day period (maximum target dosage of added fat, protein, and carbohydrate at day 1 was 0.3 g, day 2 was 0.6 g, and day 3 was 0.9 g per 100 ml breast milk), as suggested by Rochow*^*2*^*. On day 4, the full amount of TF for each macronutrient will be prescribed; this day will be marked the starting point of the intervention period. Feeding route and quantity advancement will comply with standard unit protocol*

After reaching 80 ml/kg/day of enteral feeding, patients will be randomized to receive SF (Bebilon HMF, Nutricia) or TF (protein: Bebilon Suplement Bialka, Nutricia; lipids: Calogen, Nutricia; carbohydrates: Polycal, Nutricia) (Fig. 1). The content of macro- and micronutrients of each product is given in Table 1.

**Table 1:** Content of the products used as standard fortification (ST) and tailored fortification (TF)

**Standard fortification**					
Bebilon HMF (Nutricia) *1 sachet (2.2 g) per 50 ml HM	**Energy (kcal/100 g)**	**Protein (g/100 g)**	**Carbohydrates (g/100 g)**	**Fat (g/100 g)**	**Osmolality (mmol/l)**
	347	25.2 *- Whey and casein hydrolysate (of cow’s milk)*	62.2 *- Lactose, 0.4* *- Polysaccharide, 51.6*	0	399
	**Minerals (per 100 g)**			**Vitamins (per 100 g)**	
	Natrium, 803 mg; potassium, 528 mg; chloride, 573 mg; calcium, 1491 mg; phosphate, 872 mg; magnesium, 115 mg; zinc, 14 mg; cuprum, 803 μg; manganum, 183 μg; selenium, 39 μg; iodine, 252 μg			A, 5275 μg; D, 115 μg; E, 60 mgα-TE; K, 144 μg; B1, 3005 μg; B2, 3922 μg; B3, 59 mgNE; pantothenic acid, 17,201 μg; B6, 2523 μg; folic acid, 688 μg; B12, 4.6 μg; biotin, 57 μg; C, 275 mg	
**Tailored fortification**					
	**Energy**	**Protein**	**Carbohydrates**	**Fat**	**Minerals (per 100 g)**
**Bebilon Suplement Bialka (Nutricia); protein supplement**	338 kcal/100 g	82.1 g/100 g *- Whey and casein hydrolysate (of cow’s milk)*	2.2 g/100 g - Lactose, 1.3 g/100 g	0.1 g/100 g *- Saturates, 0.1 g/100 g*	Natrium, 776 mg; potassium, 1226 mg; chloride, 66 mg; calcium, 524 mg; phosphate, 516 mg; magnesium, 46 mg; manganum, 210 μg; selenium, 27 μg
**Polycal (Nutricia)**	384 kcal/100 g (1 scoop = 5 g = 19 kcal)	0	- Maltodextrin - Lactose, < 30 mg/100 g	0	0
**Calogen Neutral (Nutricia)**	450 kcal/100 ml	0	0.1 g/100 ml	Total, 50 g/100 ml *- Saturates, 5.3 g/100 ml* *- Monounsat, 30.4 g/100 ml* *- Polyunsat, 14.3 g/100 ml* *- Ration Ω6/Ω3, 5.1*	0

*Monounsat* manosaturates, *Polysat* polysaturates

Milk fortification is routinely done twice a day (at 8 am and at 8 pm) for each following 12-h nursing shift (Fig. 1). For the purpose of the study, TF will be integrated in this schedule and performed by experienced registered nurses.

An experienced laboratory technician will perform milk analysis in the neonatal intensive care unit (NICU) research laboratory at Princess Anna Mazowiecka Hospital thrice per week at 10:00 am (Monday/Wednesday/Friday) from batches collected from the two previous days. A 10-ml aliquot from each batch of native breast milk will be used for macronutrient analysis (Miris® HMA) as per protocol (Fig. 1). The remaining batch will first be fortified with the routine fortifier. Macronutrient analysis will determine how much extra fat, protein, or carbohydrate is needed in the batch to obtain final target fortified breast milk (FBM).

Milk samples from other sites will be delivered by medical transport in secure freezing containers at 8:00 am. The mean of three measurements per batch (3 × 2–3 ml) will be used to calculate the required amount of extra fat, protein, and carbohydrate for the following 3 days of fortification using a predefined Excel spreadsheet (Microsoft Inc., Redmond, Washington). Milk analysis will be performed in both treatment arms; however, only the intervention group will receive TF. Results, together with required amounts of macronutrients, will be emailed to the participating centres before noon the same day.

The desired macronutrient concentration in breast milk will be 4.4 g/100 ml of fat, 3 g/100 ml of protein, and 8.8 g/100 ml of carbohydrate in order to meet the European Society for Paediatric Gastroenterology, Hepatology, and Nutrition (ESPGHAN) guidelines (6.6 g/kg/day of fat, 4.5 g/kg/day of protein, and 13.2 g/kg/day of carbohydrate) assuming an intake of 150 ml/kg/day.

Target fortification (TF) will be done in 3 steps:

1. Determination of macronutrient concentration in own OMM/HDM
2. SF: human milk fortifier, HMF Nutricia
3. TF: adding fat, protein, and/or carbohydrate to achieve target levels of macronutrients

In cases where a macronutrient component after SF will exceed the target value, only the other deficient macronutrient components will be adjusted.

To enhance feeding tolerance at the start of the intervention, TF will be gradually introduced in a stepwise manner over a 3-day period (maximum target dosage of added fat, protein, and carbohydrate will be as follows: at day 1, 0.3 g; day 2, 0.6 g; and day 3, 0.9 g per 100 ml breast milk), as suggested by Rochow et al. [12]. On day 4, the full amount of target fortification for each macronutrient will be prescribed; this day will be marked the starting point of the intervention period. Patients will be fed every 3 h via a gastric tube by registered nurses. Starting from 33 weeks of post-conceptional age (PCA), non-nutritive sucking stimulation will be initiated by occupational therapists. At approximately 34 weeks of PCA, infants will be transitioned to bottle feeding. When breastfeeding is established, patients will receive TF as one or two bottle feeds.

As a safety assessment to ensure that an appropriate amount of fortifier is added, osmolality of unfortified and FBM samples will be measured 3320 with the use of Micro-Osmometer; Advance Instruments, Norwood, MA. Bedside nurses will be then informed whether the osmolality of FBM is within the acceptable target range (400–480 mOsmol/kg) before the milk administered during the next 12-h shift. Osmolality lower or higher than the defined target range will be considered as a sample preparation error of fortification, and a new batch of breast milk will be prepared. Prescription of TF will be completed before noon. The attending physician will then approve these prescriptions. Subsequently, individual additives will be provided by nutrition services. Bedside nurses will prepare batches of FBM including the additives for target fortification and divide it into single feeding portions to be given to infants. Blood urea nitrogen will be used as a surrogate for protein nutriture and will be monitored weekly [16].

The intervention will continue until 37 weeks of post-conception age or hospital discharge. Parents, attending physicians, and outcome assessors will be blinded to the intervention.

#### Criteria for discontinuing or modifying allocated interventions {11b}

The following are the criteria for discontinuing allocated intervention:

- Sepsis
- NEC
- Withdrawal of parental/guardian consent
- Poor feeding tolerance defined as increasing abdominal distension > 2 cm between inter-observer measurement and/or regurgitations after feeding > 3 feeds per day

#### Strategies to improve adherence to interventions {11c}

Medical notes (MN) of infants included in the study will be visibly marked to promote adherence to the study protocol. A flowchart explaining inclusion, exclusion, and discontinuation criteria will be available in the patient’s MN.

#### Relevant concomitant care permitted or prohibited during the trial {11d}

Participants should continue to receive standard neonatal care. Interventions aimed at improving weight gain such as increased daily intake (> 160–170 ml/kg/day) or increased dosing of vitamin D (> 1000 IU/l) or prescription of milk formula will be forbidden.

#### Provisions for post-trial care {30}

The standard of care provided in participating units is high. Participating in the clinical trial will not pose any direct benefit related to extra medical care or higher standard of care during hospitalization. Ancillary care (medical care provided to clinical trial participants during a trial, which is not related to the research question) will be thus very limited. For example, more frequent contact with the attending neonatologist and neonatal nurse may be perceived as beneficial by the parents (more occasions to ask questions, also those not related to the trial). Similarly, follow-up visits and the opportunity to meet an experienced psychologist may become an opportunity to discuss parental concerns not related to our research question. Apart from out-patient follow-up appointments (as per protocol), we do not plan any additional post-discharge care. In our experience, both these factors are perceived as benefits by the participating families and contribute to their decision to participate in research projects. However, during our introductory meeting with the parents of an eligible infant, we always emphasize that the decision not to participate will not influence standard of care provided to their child in any way.

### Outcomes {12}

#### Primary outcome

Weight gain velocity (grammes per week) will be measured daily starting from the day infants regain their birth weight up to 4 weeks, then weekly until discharge. Length and head circumference will be measured in centimetres weekly until discharge.

#### Secondary outcomes

1. Growth (weight, length, head circumference) will be assessed at discharge and at 4 months of corrected age.
2. Body composition (percentage of body fat, fat mass, fat-free mass) will be measured at discharge and at 4 months corrected age by air plethysmography (PeaPod).^1^
3. Neurodevelopmental outcome:
  12 months corrected age: BSID-III (Bayley Scale of Development Third Edition); the following stratification of index composite scores will be used in our study:
    < 70 (> 2 SD below the mean)—severe impairment
    70–84 (> 1 SD below the mean)—mild impairment
    ≥ 85—normal development
  3.5 years of corrected age: BSID and WPSSI-IV (Wechsler Preschool and Primary Scale of Intelligence, Fourth Edition IV).
4. Behavioural problems: parental assessment by CBCL (Child Behaviour Check List) at 3.5 years corrected age.
5. Feeding tolerance under whole period of fortification.
6. Morbidity: incidence of necrotizing enterocolitis (NEC), retinopathy of prematurity (ROP) and bronchopulmonary dysplasia (BPD), intraventricular haemorrhage (IVH), periventricular leukomalacia (PVL), sepsis, and pneumonia. Definitions provided below.

##### Definitions

- Feeding tolerance: defined as haemorrhagic residuals or vomiting bile until pathological causes are ruled out (intestinal obstruction or ileus) [17]. Gastric residuals and abdominal girth will not be checked routinely. Isolated green or yellow residuals will be considered unimportant.
- NEC (necrotizing enterocolitis): stage II or III. Stage II requires clinical manifestations of distended abdomen and radiological verification (intramural and/or portal gas). Stage III requires findings like in stage II and more severe clinical symptoms (shock, need for respirator). In surgery verified cases, no radiological verification is needed [18].
- ROP (retinopathy of prematurity): stages I to V, diagnosed by an ophthalmologist according to international criteria [19].
- BPD (bronchopulmonary dysplasia): need for oxygen, continuous positive airway pressure (CPAP), or mechanical ventilation at 36 + 0 weeks gestational age [20].
- IVH (intraventricular haemorrhage) [21].
- PVL [21].
- Early- and late-onset sepsis defined as a positive blood or cerebral fluid culture at less or more than 72 h of age, respectively [22].

Feeding tolerance has been chosen as an efficacy and harm outcome in order to evaluate whether TEN may impact milk absorption in the intervention group. Exclusive breastfeeding decreases the risk of NEC; however, the impact of TEN on the risk of developing NEC has not yet been studied. Blood culture confirmed early-onset sepsis (EOS) is a known risk factor for delayed implementation of EN and can impact the risk of late-onset sepsis (LOS). Diagnosis of EOS and LOS can be associated with intermitted withdrawal of enteral nutrition, BPD, IVH, and PVL which are markers of neonatal morbidity; hence, we decided to include them in our analysis.

Time schedule of enrolment, interventions (including any run-ins and washouts), assessments, and visits for participants is presented in Table 2.

**Table 2:**
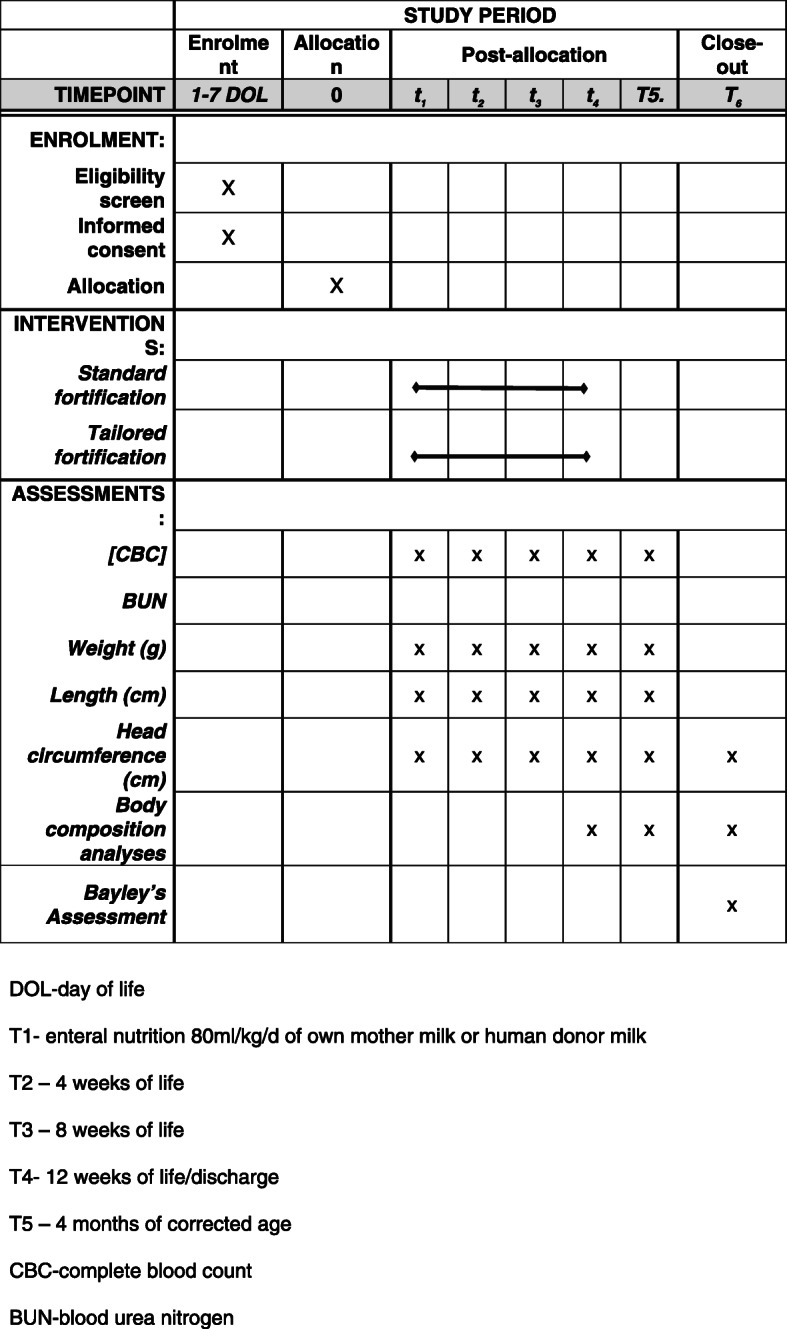
Enrolment, interventions, and assessments schedule

*DOL* day of life, *T1* enteral nutrition 80 ml/kg/day of own mother’s milk or human donor milk, *T2* 4 weeks of life, *T3* 8 weeks of life, *T4* 12 weeks of life/discharge, *T5* 4 months of corrected age, *CBC* complete blood count, *BUN* blood urea nitrogen

### Participant timeline {13}

It is provided in Table 2.

### Sample size {14}

The sample size required to compare two means in two-sided equality test was estimated based on results from a prior double blind, randomized clinical trial, investigating the effect of TG vs SF of breast milk on the changes of anthropometric parameters and body composition in preterm children [23, 24]. It was determined that a mean difference of weight gain 1.9 g/kg/day between groups would be clinically important and feasible during intervention.

The following assumptions were made for the calculation: type I error (α) 5%, power 80%, equal sample sizes in both groups, the mean weight gain in the standard fortification group 19.3 g/kg/day, and the mean weight gain in the target fortification group 21.2 g/kg/day. To account for the higher uncertainty in measured weight gain due to differences between the studied and the quoted trial population, standard deviation value taken from the prior trial was increased by 50% to 3.75.

An estimated minimum size of each group is 68. Accounting for a presumed 20% attrition rate, due to potential dropouts, deviations from the protocol, and loss to follow-up, a minimum sample size required was estimated at 156 infants or 78 infants per treatment arm.

However, in order to detect a difference of 5 points in cognitive score of Bayley-III (secondary outcome) at 12 months of age (mean of 100 points, standard deviation (SD) 12) between the study groups with a power of 80% and α = 0.05, we have decided to increase the sample to 91 infants in each study group.

In summary, allowing for 10% of loss to follow-up, the target number of 200 premature infants will be recruited.

### Recruitment {15}

**Fig. 2:**
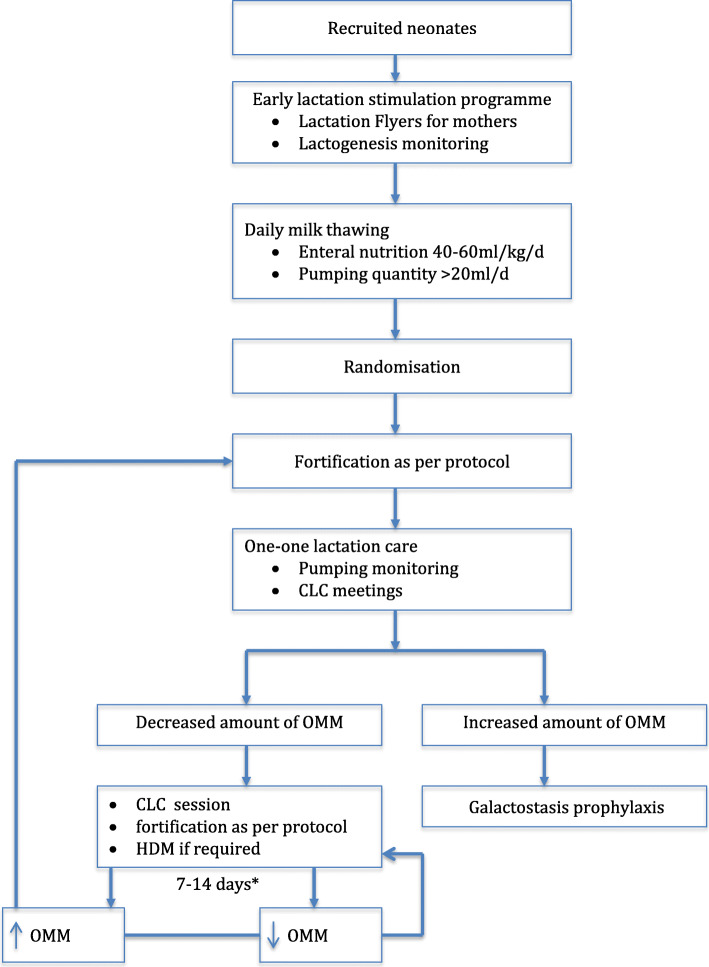
Early lactation stimulation programme. *If OMM < 20 ml/day, cessation of HMA until improvement of milk supply. OMM, own mother’s milk; CLC, certified lactation consultant; HDM, human donor milk; HMA, human milk analyses

The study will continue until the minimum of 200 valid observations are collected in every arm. As part of the antenatal consult, women with threatened preterm labour will be scheduled a short meeting with a member of the recruitment team. During this appointment, they will be offered participation in the trial. In order to increase participant enrolment, a second patient screen will be carried out by medical staff during admission to the NICU. The enrolment period will extend over 18 months. Recruitment rates will be monitored monthly. In return, women will be offered additional breastfeeding support by a certified lactation consultant (lactation stimulation programme, Fig. 2).

### Assignment of interventions: allocation

#### Sequence generation {16a}

The allocation sequence will be computer-generated by a statistical team member who will inform the researchers about the designated study group. Block randomization with stratification by delivery mode will be implemented. Patients will be randomly assigned to standard or tailored enteral nutrition fortification in 1:1 ratio. The block size will be variable and concealed until primary endpoint analyses.

#### Concealment mechanism {16b}

A member of the recruitment team will approach caregivers within the infant’s first 7 days of life. He or she will explain the study and obtain written consent for participating in the trial. Following this, the patient’s medical record number will be registered on a secure web-based platform and demographic data will be recorded. A study number together with the allocated treatment will be assigned by the platform.

#### Implementation {16c}

A member [a physician not involved in the patients care] of the research team will prescribe the allocated fortification on the patient’s drug chart. Milk fortification is routinely done twice a day (at 8 am and at 8 pm) for each following 12-h nursing shift (Fig. 1). For the purpose of the study, TF will be integrated in this schedule and performed by experienced registered nurses (RNs). The intervention will be performed by a RN blinded to treatment allocation. Patient’s data along with the result of the allocation will be sent to the statistical team. The randomization list will remain with the statistical team for the whole duration of the study.

All randomized infants who are prematurely discontinued from study will be considered *off study intervention/on study* and will follow the same schedule of events as those infants who continue study treatment. All of these infants will be followed through 36 months as scheduled. Once an infant is enrolled or randomized, the study site will make every reasonable effort to follow the infant for the entire study period. Participants may withdraw from the study for any reason at any time.

### Assignment of interventions: blinding

#### Who will be blinded {17a}

The bedside nurse, treating physicians, clinical psychologist, and data analysts will be blinded to the treatment allocation. Milk fortification will be performed in the milk bank by an experienced RN. Prepared milk portions will be transported to unit. Feeding portions from both treating arms will not differ in colour and structure.

#### Procedure for unblinding if needed {17b}

If unblinding is necessary, the actual allocation will only be disclosed to the treating physician. The investigator must report all code breaks (with reason) as they occur on the corresponding confidential recruitment file (CRF) page. Unblinding should not necessarily be a reason for study drug discontinuation.

### Data collection and management

#### Plans for assessment and collection of outcomes {18a}

##### Primary outcome

Weight gain velocity will be measured starting from the day infants regain their birth weight up to 4 weeks. Length and head circumference will be measured weekly until discharge and at 4 months of corrected age.

##### Secondary outcomes

1. Neurodevelopmental outcome:
  12 months corrected age: BSID-III (Bayley Scale of Development Third Edition);
  3.5 years of corrected age: BSID and WPSSI-IV (Wechsler Preschool and Primary Scale of Intelligence, Fourth Edition IV).
2. Behavioural problems: parental assessment by CBCL (Child Behaviour Check List) at 3.5 years corrected age.
3. Feeding tolerance under whole period of fortification.
4. Morbidity: NEC, ROP, BPD, IVH, PVL, sepsis, and pneumonia. Definitions provided in the “Outcomes {12}” section.

Time schedule of enrolment, interventions (including any run-ins and washouts), assessments, and visits for participants is presented in Table 2.

All data will be collected by the study personnel using electronic case report forms (e-CRF), developed specifically for the study. All data will be stored on a password-secured website. An introduction training session will be held at the beginning of the study for all study personnel at each study site.

#### Plans to promote participant retention and complete follow-up {18b}

All randomized infants who are prematurely discontinued from study drug will be considered *off study drug/on study*. They will follow the same participant timetable as those infants who continue study treatment. All of these infants will be followed through 36 months as scheduled.

Once an infant is enrolled or randomized, the study site will make every reasonable effort to follow the infant for the entire study period. It is projected that the rate of loss to follow-up on an annual basis will be at most 20%. Each study site staff will develop and implement local standard operating procedures to achieve this level of follow-up.

Participants may withdraw from the study for any reason at any time. The investigator also may withdraw participants from the study in order to protect their safety.

#### Data management {19}

All data will be entered electronically. Data integrity will be enforced through a variety of mechanisms. Referential data rules, valid values, range checks, and consistency checks against data already stored in the database (i.e. longitudinal checks) will be supported. Modifications to data written into the database will be documented through either the data change system or an inquiry system. Data entered into the database will be retrievable for viewing through the data entry applications. The type of activity that an individual user may undertake will be regulated by privileges associated with his/her user identification code and password. A complete backup of the database will be performed twice a month.

#### Confidentiality {27}

Complete patient and study information will be stored on a secure, password-protected, web-based platform. Only researchers involved in the study will be provided with a personalized login and password to access the study information. The statistical team will not have access to sensitive data such as date of birth, address, and contact details. All records containing the above patient details and relevant medical history will be stored separately in a locked file cabinet.

#### Plans for collection, laboratory evaluation, and storage of biological specimens for genetic or molecular analysis in this trial/future use {33}

We do not plan to perform any genetic or molecular analysis in this trial. Maternal milk samples will be labelled and stored in a separate refrigerator dedicated for biological material in the NICU. All left over samples of maternal milk will be destroyed as per hospital protocol for biohazard waste.

### Statistical methods

#### Statistical methods for primary and secondary outcomes {20a}

Baseline characteristics will be presented for all included neonates in intention-to-treat and per-protocol analysis sets, by treatment group. Dichotomous variables will be presented as frequencies, categorical variables as median and interquartile ranges, and continuous variables as mean and standard deviations, along with 95% CI. In cases of missing data, i.e. due to adverse events or dropouts, in follow-up analysis, a weighted average taking account of changes in the gender ratio will be applied. Continuous variables will be tested against normality of distribution and the equality of variances between groups. For continuous variables distributed normally, the differences in means will be tested using *t* test; for continuous variables not distributed normally and for categorical variables between groups, comparison will be performed using the Mann-Whitney *U* test. The primary outcome will be assessed with one-tailed unpaired two-sample *t* test for noninferiority. Secondary and safety outcomes will be tested for two-sided superiority. Proportions of dichotomous variables will be tested using chi-square or Fisher’s exact test, whichever appropriate. In addition, the risk ratio (RR), the odds ratio (OR), and the number needed to treat will be assessed. For secondary outcomes, the difference will be considered significant when *p* value calculated in statistical tests will be < 0.05 or when the 95% CI for the mean difference will not include 0, or 1 in case of RR or OR.

#### Interim analyses {21b}

Due to the short duration of recruitment and low risk of potentially serious outcomes, an interim analysis will not be performed.

#### Methods for additional analyses (e.g. subgroup analyses) {20b}

Given the homogeneity of our study groups, we do not plan to conduct any subgroup analyses.

#### Methods in analysis to handle protocol non-adherence and any statistical methods to handle missing data {20c}

Linear regression will be used to fill missing data.

In addition, a sensitivity analysis using (1) missing as missing (i.e. no imputation) for dropouts and (2) last observation carried forward (LOCF method) will be performed for the primary endpoint to explore the impact of missing data after a subject drops out. All participants will be included in the intention-to-treat (ITT) analysis, regardless of adherence. In addition, per-protocol (PP) analysis of patients with complete observations will be performed to estimate the effect of missingness or protocol deviations on the statistical analysis.

### Plans to give access to the full protocol, participant-level data, and statistical code {31c}

Access to the full protocol and participant-level data will be given on request from the corresponding author.

### Oversight and monitoring

#### Composition of the coordinating centre and trial steering committee {5d}

The coordinating centre is the Department of Neonatology at the Medical University of Warsaw where the principal investigator (PI), Joanna Seliga-Siwecka, is affiliated. The steering committee consists of the PI (JS-S), Anna Chmielewska, and Katarzyna Jasińska. The committee is responsible for communication and trial coordination. Data management team consists of Jakub Rutkowski, an experienced biostatistician, and the PI.

#### Composition of the data monitoring committee, its role and reporting structure {21a}

##### Data monitoring

In light of the short duration of the study and minimal risk to participating neonates, we have decided not to form a formal data monitoring committee (DMC).

#### Adverse event reporting and harms {22}

##### Harms

We will define an adverse event as any untoward medical occurrence in a subject without regard to the possibility of a causal relationship. Adverse events will be collected after the subject has provided consent and enrolled in the study. All adverse events occurring after entry into the study and until hospital discharge will be recorded. An adverse event that meets the criteria for a serious adverse event (SAE) between study enrolment and hospital discharge will be reported to the local Ethical Committee. A serious adverse event for this study is any untoward medical occurrence that is believed by the investigators to be causally related to study intervention and results in any of the following: life-threatening condition (that is, immediate risk of death), severe or permanent disability, and prolonged hospitalization. Serious adverse events occurring after a subject is discontinued from the study will *not* be reported unless the investigators feel that the event may have been caused by the study drug or a protocol procedure. The following AE will be reported: feeding intolerance defined as vomiting and emesis, abdominal distention, and gastric residuals within 7 days after the introduction of TEN. SAE will include necrotizing enterocolitis and gastrointestinal obstruction occurring after the introduction of TEN. Adverse events will be collected within the e-CRF and reported in trial publications.

#### Frequency and plans for auditing trial conduct {23}

##### Auditing

Auditors appointed by the foundation will audit patient recruitment bimonthly.

They will audit the overall quality and completeness of the data, examine source documents, interview investigators and coordinators, and confirm that the clinical centre has complied with the requirements of the protocol. The monitors will verify that all adverse events were documented in the correct format and are consistent with protocol definition.

#### Plans for communicating important protocol amendments to relevant parties (e.g. trial participants, ethical committees) {25}

Any modifications to the protocol which may impact on the conduct of the study and potential benefit of the patient or may affect patient safety, including changes of study objectives, study design, patient population, sample sizes, study procedures, or significant administrative aspects, will require a formal amendment to the protocol. Such will need to be approved by the Research Ethics Board of the Medical University of Warsaw prior to implementation and notified to the health authorities in accordance with local regulations.

#### Dissemination plans {31a}

We plan to publish the full protocol, so it shall be widely available due to open access. We plan to submit our findings to international peer-reviewed journals (paediatric, gastroenterology, nutrition). Abstract will be submitted to local and international conferences.

## Trial status

Protocol version 3: 22 September 2020

Recruitment start: 01 June 2019

Approximate compliment: 31 December 2022

## Data Availability

Deidentified participation data will be available on request from the corresponding author. Data reuse will be permitted for systematic reviews and meta-analysis. We do not have any contractual agreements which limit access to data to declare. The datasets used and/or analysed during the current study will be available from the corresponding author on reasonable request.

## Abbreviations

EUGR: Extrauterine growth restriction
VLBW: Very low birth weight
NEC: Necrotizing enterocolitis
OMM: Own mother’s milk
DHM: Donor human milk
SF: Standard fortification
BUN: Blood urea nitrogen
TF: Tailored/targeted fortification
BSID-III: Bayley Scale of Development Third Edition
WPSSI-IV: Wechsler Preschool and Primary Scale of Intelligence, Fourth Edition IV
CBCL: Child Behaviour Check List
ROP: Retinopathy of prematurity
BPD: Bronchopulmonary dysplasia
GA: Gestational age
MRN: Medical record number
NICU: Neonatal intensive care unit
FBM: Final target fortified breast milk
ESPGHAN: European Society for Paediatric Gastroenterology, Hepatology, and Nutrition
PCA: Post-conceptional age
MN: Medical notes
PVL: Periventricular leukomalacia
CPAP: Continuous positive airway pressure
IVH: Intraventricular haemorrhage
SD: Standard deviation
RNs: Registered nurses
CRF: Confidential recruitment file
RR: Risk ratio
OR: Odds ratio
LOCF method: Last observation carried forward
DMC: Data monitoring committee
SAE: Serious adverse event

## Footnotes

1 PeaPod device is not yet available at the study site. Additional financing has been applied for (cost of PeaPod: approx. 600,000 PLN), and recruitment will be initiated before any decisions are available. Thus, there is a risk that body composition will not be determined in this study. This information will be given to parents of eligible infants at study entry.
